# Latent State–Trait and Latent Growth Curve Modeling of Smooth Pursuit Eye Movements

**DOI:** 10.1111/psyp.70299

**Published:** 2026-04-24

**Authors:** Celina Kullmann, Ulrich Ettinger, Kaja Faßbender

**Affiliations:** ^1^ Department of Psychology University of Bonn Bonn Germany

**Keywords:** latent growth‐curve, latent state–trait, reliability, smooth pursuit eye movements

## Abstract

Smooth pursuit eye movement (SPEM) performance has previously been shown to have good reliability. While quantifying the relative amounts of reliable trait and state influences on SPEM is relevant to different lines of research including individual differences, clinical and experimental research, this has not yet been done. Here, we apply latent state–trait (LST) theory to SPEM for the first time to examine reliability and to explicitly decompose trait and situational variance. SPEM tasks with sinusoidal and triangular movement patterns were performed by *N* = 163 healthy participants at three measurement occasions. LST and latent growth curve (LGC) modeling was used to calculate model‐based reliability and to distinguish reliable trait variance (consistency) and variance due to influences of the situation and of the person × situation interaction (occasion specificity). We found mostly excellent reliabilities (0.86–0.98), except for the intra‐individual standard deviation of root mean square error (RMSE) in both SPEM tasks where reliability was good (0.70–0.74). Consistencies and occasion specificities indicated that a higher proportion of variance was due to trait influences (62% on average) than due to situational influences (26% on average). There were mostly no changes on trait level over time. We conclude that SPEM performance is highly reliable and mainly reflects relatively stable trait components, but is also characterized by substantial state influences. Overall, these findings further support the use of SPEM in individual differences studies. However, potential state influences should be considered more explicitly in future studies examining SPEM.

## Impact Statement

This is the first study quantifying state and trait components of smooth pursuit eye movements (SPEM) by applying latent‐state trait (LST) theory. SPEM performance was highly reliable and characterized by larger trait than state components across all performance parameters, indicating that SPEM are suitable for individual differences research. While this pattern was roughly similar to what was found previously for saccadic eye movements, state influences on SPEM were more pronounced.

## 1 Introduction

Eye movements have long been studied in experimental, clinical, and, more recently, individual differences research (Bargary et al. 2017; Leigh and Zee 2015; Smyrnis et al. 2019). One type of eye movements are smooth pursuit eye movements (SPEM), which serve to hold the image of a small, slowly moving stimulus on the fovea centralis (Leigh and Zee 2015). SPEM involve both sensorimotor and cognitive processes, and can be examined using different patterns of target movements in the experimental setting, such as targets moving at constant velocity (triangular pattern) or at sinusoidally varying velocity (sinusoidal pattern) (Lencer and Trillenberg 2008). There are different parameters for quantification of SPEM performance, such as velocity gain as a specific performance measure, as well as the root‐mean square error (RMSE) and total saccade frequency as more global performance measures (Smyrnis 2008).

While it is common practice to report psychometric properties like reliability of individual differences measures such as self‐report questionnaires, this is not yet routinely done for cognitive‐behavioral measures (Parsons et al. 2019). Reliability refers to the proportion of variance that is free from measurement error in classical test theory (CTT; Novick 1966). Reliability coefficients ≥ 0.80 are usually interpreted as excellent, ≥ 0.60 as good, and ≥ 0.40 as moderate (Hedge et al. 2018). It has been argued that reliability is crucial to derive accurate conclusions particularly in individual differences studies (Hedge et al. 2018; Parsons et al. 2019). Previous reliability studies of SPEM performance (overview in Table 1) indicated mostly good reliability, although coefficients often ranged from low to high across studies. It has been argued that differences in reliability might partly be due to differing task difficulty as shown by higher reliability in conditions with faster compared to slower target velocities (Ettinger et al. 2003; Schröder et al. 2021).

**TABLE 1:** Reliability of smooth pursuit performance in previous studies.

	Temporal stability	Internal consistency			References
	Test–retest	Split‐half	Odd‐even	Cronbach's α	
Sinusoidal SPEM					
Velocity gain	ICC = 0.50–0.90 *r* = 0.51–0.79	ICC = 0.56–0.93 *r* = 0.57–0.92			^a^, ^b^, ^c^, ^d^
RMSE	ICC = 0.30–0.57 *r* = 0.42–0.87	ICC = 0.42–0.72 *r* = 0.43–0.76			^d^, ^e^, ^f^
Saccade frequency	ICC = 0.41–0.90 *r* = 0.41–0.80	ICC = 0.30–0.68 *r* = 0.30–0.70			^a^, ^d^, ^g^
Triangular SPEM					
Velocity gain	ICC = 0.10–0.86 *r* = 0.11–0.86 *ρ* = 0.88	*ρ* = 0.89	*ρ* = 0.96	*α* = 0.83–0.88	^h^, ^i^
RMSE	ICC = 0.80 *r* = 0.81 *ρ* = 0.79	*ρ* = 0.86	*ρ* = 0.91		^h^
Saccade frequency	ICC = 0.34–0.93 *r* = 0.42–0.94 *ρ* = 0.78–0.83	*ρ* = 0.87–0.89	*ρ* = 0.93	*α* = 0.34–0.88	^h^, ^i^

*Note:* Reliabilities in terms of temporal stability and internal consistency from previous studies are given regarding smooth pursuit velocity gain, root mean square error (RMSE) and saccade frequency. Reliabilities for saccade frequency include reliability estimates for specific types of saccades, such as for catch‐up saccades, anticipatory saccades, etc. Only reliabilities obtained in standard sinusoidal and triangular smooth pursuit eye movement (SPEM) tasks are reported in the table (e.g., *no* blanking tasks, step‐ramp tasks, trapezoidal tasks, SPEM tasks with structured background, or with trunk rotation). Moreover, only reliabilities referring to healthy individuals are included.

Abbreviations: *α*, Cronbach's alpha; *ρ*, Spearman correlation; ICC, intraclass correlation; *r*, Pearson correlation.

^a^ Flechtner et al. (2002).

^b^ Majcen Rosker et al. (2021).

^c^ Majcen Rosker et al. (2022).

^d^ Schröder et al. (2021).

^e^ Gooding et al. (1994).

^f^ Iacono and Lykken (1981).

^g^ Rea et al. (1989).

^h^ Bargary et al. (2017).

^i^ Ettinger et al. (2003).

In addition to reliability, it is crucial to investigate trait and state influences on SPEM performance. For individual differences research, knowledge about whether SPEM performance reflects rather stable traits or is more state dependent is particularly important to further improve the understanding of associations between SPEM performance and other constructs. Such constructs may be personality correlates such as schizotypy, demographic variables such as age or sex, but also performance parameters from other oculomotor tasks (e.g., Bargary et al. 2017; Coors et al. 2021; Lenzenweger and O'Driscoll 2006). In clinical research, SPEM have long been regarded as an endophenotype of schizophrenia (Gottesman and Gould 2003) based on robustly reduced performance in individuals with schizophrenia and unaffected relatives compared to healthy controls (Calkins et al. 2008; O'Driscoll and Callahan 2008). Examination of trait and state influences is crucial for this line of research as endophenotypes not only require reliable measurement (Liu and Gershon 2024), but also relative state‐independence (Glahn et al. 2014; Gottesman and Gould 2003; however, see Liu and Gershon 2024). Moreover, knowledge of potential state influences on SPEM might aid to better account for such influences in future studies where they may be considered confounding factors, particularly in experimental research.

So far, the question regarding relative amounts of trait and state variance in SPEM performance has been addressed only indirectly without formally modeling these sources of variance. It has been argued that SPEM performance is trait‐like in nature, as indicated by mostly high reliability (Table 1) and the prevailing absence of changes at group level between repeated measurements in healthy individuals (Ettinger et al. 2003; Flechtner et al. 2002; Gooding et al. 1994; Schröder et al. 2021), relative temporal stability in individuals with schizophrenia mostly independent of clinical status (Flechtner et al. 2002; Gooding et al. 1994), as well as the presence of genetic influences (Bargary et al. 2024; Lencer et al. 2017) showing that SPEM performance has substantial heritability (Blekher et al. 1997; Katsanis et al. 2000). However, there are also state influences on SPEM, such as performance declines with sleepiness, and in its extreme form sleep deprivation (De Gennaro et al. 2001; Meyhöfer et al. 2017). While research implicates potentially stronger trait than state influences on SPEM performance, it is crucial to explicitly dismantle and quantify both sources of variance. However, such research is still lacking.

To formally model state and trait components, structural equation modeling (SEM) based on latent state–trait (LST) theory can be applied (Geiser et al. 2015; Steyer et al. 1999, 2015). Extending CTT, it is assumed in LST theory that psychological measurements are not only influenced by measurement error and characteristics of the individual but also by situational factors. LST modeling allows us to differentiate and quantify the amount of variance due to trait influences (consistency) and the amount of variance due to influences of the situation and person × situation interaction (occasion specificity). Model‐based reliability can be determined as the sum of consistency and occasion specificity. Latent growth curve (LGC) modeling further extends LST models, allowing us to quantify individual differences in trait change over time (Geiser et al. 2015). Crucially, while situational effects may be interpreted as measurement error in CTT in longitudinal designs, this may be prevented when applying LST theory due to the specific variance decomposition (Steyer et al. 2015).

LST theory has successfully been applied in many studies already (for an overview, see Geiser and Lockhart 2012, appendix B), mainly focusing on questionnaire data (e.g., Scarpato et al. 2021; Steyer et al. 1992). Moreover, there are LST studies on cognitive performance (e.g., Danner et al. 2011; Faßbender et al. 2023). Meyhöfer et al. (2016) conducted the first LST study on eye movements. They found mostly good to excellent reliability of antisaccade and prosaccade task performance, as well as mostly moderate to excellent reliability of memory‐guided saccades, while variables capturing intra‐individual variability of performance often showed lower reliability. Moreover, results suggested that saccadic eye movement parameters are trait‐like and situational influences were generally low. A similar pattern was reported in a later LST study focusing on inhibitory control tasks, including antisaccades and prosaccades, although slightly higher reliability was found (Faßbender et al. 2023). In contrast to Meyhöfer et al. (2016), Faßbender et al. (2023) also applied LGC modeling and observed significant trait changes over time in antisaccade and prosaccade performance.

However, LST/LGC modeling has not yet been applied to SPEM. This kind of modeling is of great interest, as it provides the opportunity to explicitly decompose and quantify trait and state influences, potentially preventing state influences from being interpreted as measurement error. This approach may not only lead to more accurate reliability estimates but also increased knowledge on trait and state characteristics of SPEM which is useful for individual differences, clinical as well as experimental research.

Here, we provide the first study applying LST modeling to SPEM. Doing so, we aimed to add to existing SPEM reliability studies by providing LST model‐based estimations and expected at least good reliability. To enhance comparability with previous studies, we also provide traditional reliability coefficients based on CTT. Moreover, we aimed to quantify the relative amounts of trait and state influences. Based on previous LST studies on eye movements, we hypothesized that SPEM performance parameters represent relatively stable traits, as reflected by higher consistency than occasion specificity. In addition, we provide exploratory analyses on potential trait changes over time based on LGC modeling.

## 2 Methods

### 2.1 Participants

We aimed to recruit a sample of 150 healthy participants (50% women, 50% men) with complete datasets, who were identical to a previous study (Faßbender et al. 2023). Inclusion criteria were age of 18–30 years, written informed consent to participate, right‐handedness, and normal or corrected‐to‐normal vision. Exclusion criteria were current medication intake (exceptions were oral contraceptives and thyroid medication), diagnosis of any current or past psychiatric condition, and color blindness. Participants received either 40€ or course credits as compensation. Ethical approval was obtained by the ethics committee of the Department of Psychology at the University of Bonn. Study aims and procedures were preregistered at https://aspredicted.org/4j6x‐vr3m.pdf.

### 2.2 Design and Procedure

Participants were recruited via advertisements distributed online and around the campus of the University of Bonn. First, participants completed an online screening questionnaire (SoSci Survey; Leiner 2019) consisting of demographic questions as well as questions regarding inclusion/exclusion criteria, including the Edinburgh Handedness Inventory (Oldfield 1971). Individuals eligible for participation were invited to our laboratory for three consecutive study sessions. Sessions were arranged to be exactly 1 week apart at the same time of day (±1 h). Each session lasted approximately 80 min in total. In the beginning of the first session, experimenters asked for verbal confirmation of inclusion/exclusion criteria. Moreover, the Ishihara test (Ishihara 1917) as a measure of color blindness was applied. Participants then performed eight oculomotor and cognitive tasks, always beginning with two variants of SPEM tasks (sinusoidal and triangular) which had an overall duration of about 3 min. SPEM tasks were presented in randomized order, yet task order was counterbalanced across participants and kept constant for each participant across all sessions. Afterwards, six inhibitory control tasks were applied, which were examined elsewhere (Faßbender et al. 2023).

### 2.3 Participant Setup and Eye Movement Recording

We used a desktop‐mounted video‐based combined pupil and corneal reflection eye‐tracker (EyeLink 1000, SR Research Ltd., Canada), with a mean accuracy of 0.25° to 0.5° and a spatial resolution of < 0.01° RMS. Monocular tracking of the right eye (horizontal and vertical position) was performed by detecting pupil position and corneal reflection via a centroid pupil‐tracking algorithm, at a sampling rate of 1000 Hz. Testing took place in a darkened room and a chinrest was used to reduce head movements during recording. Tasks were written in Experiment Builder (version 1.10, SR Research Ltd., Canada) and presented on a 24″ BenQ flat‐screen monitor (screen dimensions: 53.1 × 29.9 cm, resolution: 1920 × 1080 px; refresh rate: 144 Hz) with an eye‐to‐monitor distance of about 70 cm.

### 2.4 Task Procedures

Prior to each SPEM task, a horizontal‐vertical 5‐point calibration with subsequent validation and a central drift correction procedure was performed (for details, see Appendix S1). Participants were instructed to follow a slowly, horizontally moving target stimulus with their eyes as accurately as possible while avoiding head movements. Instructions were shown at central position (0°, 0°) as white text (RGB 255, 255, 255) on a black background (0, 0, 0). Participants were asked to repeat task instructions in their own words to ensure task comprehension.

In both tasks, the target stimulus was a light gray (192, 192, 192) disk (diameter = 0.34°; stroke width = 0.11°) on black background (0, 0, 0). Target movement always began with a movement from central position (0°, 0°) to the right. The target then continued to move on the horizontal plane within an amplitude of about ±9.80°. Vertical position remained constant throughout both tasks (0°). The sinusoidal SPEM task took 76.57 s, and a sinusoidal movement pattern with a frequency of 0.4 Hz was used. The triangular SPEM task took 91.50 s, and a triangular movement pattern with a constant target velocity of 13°/s was used.

### 2.5 Data Processing

Eye movement recordings were visually screened using Data Viewer software (version 4.4.1, SR Research Ltd., Canada) and files with noisy data segments were manually tagged (by CK). Next, a trained student assistant visually screened all tagged files again and compiled a list of time stamps corresponding to noisy segments. This list was then rechecked by CK and used as the basis to exclude noise segments.

We used MATLAB R2024b (The Math Works Inc 2024) for pre‐processing and for calculation of dependent variables. First, previously defined noise segments as well as data prior to the first direction reversal point and data after the last reversal point were excluded. Thus, a total of 60 half‐cycles were used in each of the two SPEM tasks, 30 of which corresponded to movements to the right and 30 to movements to the left. Eye movement velocity and acceleration were determined using a 17‐sample model, following guidelines provided by SR Research. For velocity gain, segments lasting ≥ 50 ms in the middle 50% of each half‐cycle were considered. Blinks, saccades, and noise segments were excluded from gain calculation. Saccades were defined based on criteria regarding amplitude (≥ 1.0°) as well as velocity and acceleration (velocity ≥ 60°/s or velocity ≥ 22°/s and acceleration ≥ 3800°/s^2^). Velocity gain was calculated as time‐weighted average ratio of eye velocity and target velocity across all samples, multiplied by 100. In addition, time‐weighted intra‐individual standard deviation of velocity gain across all samples was examined. RMSE was determined as the square root of the mean squared angular distances between eye and target position across all samples. Moreover, intra‐individual standard deviation of RMSE across half‐cycles was determined. Again, both measures were time‐weighted. Blinks, saccades around blinks, and noise were excluded from RMSE calculation. Saccade frequency was defined as the number of saccades per second. Saccades were determined based on saccade criteria specified above, without considering saccades around blinks and during noise segments.

All dependent variables were calculated as aggregated measures for each measurement occasion (for temporal stability), test set (for SEM, i.e., LST and LGC modeling) and half‐cycle (for internal consistency). Test sets were generated using an odd‐even classification of cycles, ensuring that both test sets contained half‐cycles with movements to the right and to the left. We determined intra‐individual standard deviation of RMSE only as aggregated measures for each occasion and test set, since calculation per half‐cycle would have required further segregation of half‐cycles into even shorter time segments.

Outlier detection and further analyses were carried out using R 4.5.1 (R Core Team 2025). Pre‐processed data as well as R scripts including a list of used R packages are available online (https://osf.io/fcmyg/overview?view_only=4d42c9ce61514669a278e7336f01009a).

Deviating from our preregistration but following recommendations from a previous review process (Faßbender et al. 2023), all participants who completed at least one task at one measurement occasion were included. Statistical analyses were performed only after deciding to also include participants with incomplete data. Participants for whom calculation of a dependent variable on a specific measurement occasion and test set was based on less than 50% of the possible time window were excluded from the respective measurement occasion for that particular variable. We applied this criterion only for datasets used for SEM analyses. For calculation of Cronbach's alpha, participants with less than nine available half‐cycles were excluded from the respective session for that particular variable. Outliers were removed prior to any analyses and were defined as values smaller or larger than four times the interquartile range (IQR) at the upper and the lower end, again deviating from our preregistration (values more or less than 3*IQR) but following recommendations from a previous review process (Faßbender et al. 2023). Comprehensive information on final sample sizes including details on data exclusion and outliers are presented online (https://osf.io/fcmyg/overview?view_only=4d42c9ce61514669a278e7336f01009a).

### 2.6 Statistical Analyses

#### 2.6.1 SEM Analyses

**FIGURE 1:**
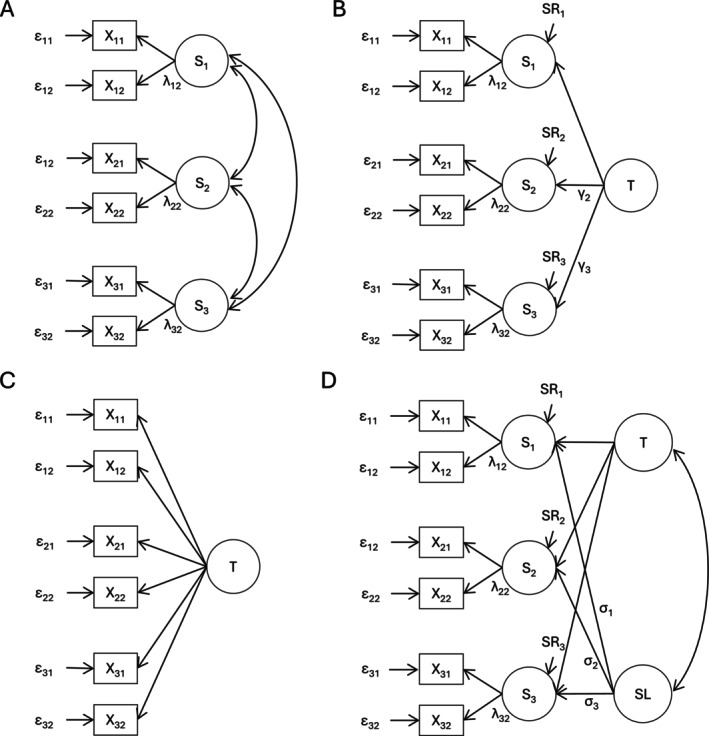
LS, LST, LT, and LGC Models. Panel A shows the latent state (LS) model with free estimation of state means. Panel B shows the latent state–trait model with free estimation of state intercepts (LST) or trait means (LST_T_). Panel C shows the latent trait model. Panel D shows the second‐order latent growth curve (LGC) model with free estimation of trait and slope means. Remaining means and intercepts were set to zero and all loadings without labels were set to 1. Double‐arrows represent correlations. All models were based on three measurement occasions i with each two test sets k. X_*ik*_ = manifest dependent variables; ε_*ik*_ = measurement error variances; S_*k*_ = latent states; SR_*k*_ = latent state residuals; *λ*_*ik*_ = state loadings; T = latent trait; *γ*_*k*_ = trait loadings; SL = latent slope factor; *σ*_*k*_ = slope loadings.

Statistical methods were similar to our previous publications on LST and LGC modeling (especially Faßbender et al. 2023; partly Meyhöfer et al. 2016). SEM based on covariance matrices of each measurement occasion (aggregated dataset per test set) was used to set up latent state (LS; Figure 1A), LST (Figure 1B), latent trait (LT, Figure 1C) and second‐order LGC models (Figure 1D). Models were set up separately for each dependent variable. We assessed each dependent variable using two test sets (*k*) at each of the three measurement occasions (*i*), as required for LST modeling (Kelava and Schermelleh‐Engel 2012). LS models were used to test for measurement invariance (MI) and homogeneity (Geiser et al. 2015), and manifest observed variables (X_*ik*_) were decomposed into measurement error variance (*ε*_*ik*_) and latent states (S_*i*_). In LST models, latent states were further decomposed into a latent trait factor (T) representing dispositional influence, and occasion‐specific state residuals (SR_*i*_) indicating influences of the situation and the person × situation interaction (Steyer et al. 1999, 2015). In LGC models, a latent slope factor (SL) was added, representing trait changes across measurement occasions (Geiser et al. 2015). Latent states, state residuals and latent slope were not considered in LT models, where manifest observed variables were decomposed into measurement error variance and a latent trait factor.

We compared models using χ^2^ difference tests under consideration of changes in the comparative fit index (ΔCFI, should be < 0.01; Cheung and Rensvold 2002; Isiordia and Ferrer 2018). In addition, we considered model fits as assessed based on *χ* ^2^ statistics (should be n.s.), and the following fit indices: CFI which should be ≥ 0.95; root mean square error of approximation (RMSEA, including confidence intervals) which should be ≤ 0.06; and standardized root mean residual (SRMR) which should be ≤ 0.08 (Beauducel and Wittmann 2005; Bentler 2007; Hu and Bentler 1999). Local fit was assessed based on the significance of single model parameters, using critical ratio statistics (c.r., = parameter estimate/standard error of this parameter; Bühner 2021).

Mardia test (Mardia 1970) was applied to test for the assumption of multivariate normality. If multivariate normality was given, parameter estimation was based on maximum likelihood (ML) estimation method. If the assumption of multivariate normality was violated, we used robust maximum likelihood estimation (MLR; Huber‐White robust standard errors; Yuan‐Bentler scaled test statistics; scaled *χ* ^2^ differences tests, Satorra and Bentler 2001). Deviating from our preregistration, we applied the Full Information Maximum Likelihood (FIML) method to account for missing observations since we also included participants with incomplete data (see above).

We began with calculating LS models. A baseline LS model without any restrictions was compared to LS models with increasing amounts of restrictions: (1) weak MI (*λ*_12_ = *λ*_22_ = *λ*_32_, i.e., equal state loadings), (2) strong MI (*λ*_12_ = *λ*_22_ = *λ*_32_; intercepts of observed variables set to zero; free estimation of factor means), and (3) strict MI (*λ*_12_ = *λ*_22_ = *λ*_32_; intercepts of observed variables set to zero; free estimation of factor means; *ε*_11_ = *ε*_12_ = *ε*_21_ = *ε*_22_ = *ε*_31_ = *ε*_32_, i.e., equal error variances; τ‐equivalence). At least strong MI is required for proper LST/LGC modeling (Geiser et al. 2015). If *χ* ^2^ difference tests indicated violation of this prerequisite, we evaluated MI based on model fit indices to ensure that the assumption of strong MI was not rejected despite good model fit. If the prerequisite of at least strong MI was still violated, we used three instead of two indicators defined by cyclic classification of SPEM cycles, thereby deviating from our preregistration but following previous recommendations (Geiser et al. 2015; Schermelleh‐Engel et al. 2003).^1^ Moreover, we compared the baseline LS model to a model with an indicator‐specific factor loading on either odd or even cycles to test for homogeneity of indicators. In case of indicator‐specific effects, indicator‐specific factors were included. LS models were only set up to test for MI and homogeneity and were not statistically compared with LST, LGC and LT models.

We proceeded with LST, LGC, and LT modeling, always aiming for the most restrictive best fitting model. Two versions of LST models were set up, which were not specified in our preregistration but recommended in a previous review process (Faßbender et al. 2023). While trait changes were allowed in LST models with free estimation of state intercepts, LST_T_ models with free estimation of trait means did not allow this. All other intercepts and means of latent variables in these models were set to zero. Quantification of trait changes was possible only in LGC models, providing exploratory analyses in addition to what we preregistered.

First, nested models were tested against one another to find the most restrictive best fitting LST, LST_T_, LGC, and LT model, respectively. In the next step, these preselected models (non‐nested models) were compared. In both steps, we first considered *χ* ^2^ difference tests to identify models fitting significantly worse compared to other models.^2^ Remaining models were then evaluated regarding model fit indices. In case there was more than one fitting model, the most restrictive of these models was selected. Regarding non‐nested model comparisons, we also considered whether LGC models indicated trait changes (slope mean) or individual differences in trait changes (slope standard deviation).

Increasing number of model restrictions were applied where applicable: (1) equal measurement error variances (*ε*_11_ = *ε*_12_ = *ε*_21_ = *ε*_22_ = *ε*_31_ = *ε*_32_), (2) equal state residuals and trait loadings fixed to 1 (SR_1_ = SR_2_ = SR_3_; *γ*_1_ = *γ*_2_ = *γ*_3_ = 1; i.e., parallelism), and (3) state loadings on manifest variables fixed to 1 (*λ*_12_ = *λ*_22_ = *λ*_32_ = 1). Regarding LGC models, we set slope loadings for the first and second measurement occasion to zero and 1, respectively (*σ*_1_ = 0, *σ*_2_ = 1). Slope loadings for the third occasion were freely estimated first, but set to fixed values if free estimation was not possible. Fixed values comprised setting loadings to values estimated in a less restrictive model, or modeling linear trait changes (*σ*_3_ = 2), trait changes becoming less over time (*σ*_3_ = 1.5) or trait changes occurring only between the first and second occasion (*σ*_3_ = 1).

Based on the most restrictive best fitting model, reliability, common consistency and occasion specificity were computed (Geiser et al. 2015; Steyer et al. 1992; Steyer and Schmitt 1990). High reliability indicates the degree to which measurements are free from measurement error and represent the sum of consistency and occasion specificity:

(1) $$\mathrm{Rel}\left(X_{\mathrm{ik}}\right)=\mathrm{Con}\left(X_{\mathrm{ik}}\right)+\mathrm{Spe}\left(X_{\mathrm{ik}}\right)$$

Consistency indicates the amount of dispositional influences, and represents the proportion of variance in manifest variables explained by trait influences in LST models:

(2) $$\mathrm{Con}{\left(X_{\mathrm{ik}}\right)}_{\mathrm{LST}}=\frac{\lambda_{\mathrm{ik}}^{2}\;\gamma_{i}^{2}\;\mathrm{Var}\left(T\right)}{\mathrm{Var}\left(X_{\mathrm{ik}}\right)}$$

In LGC models, consistency is the proportion of variance in manifest variables explained by influences of trait and trait change:

(3) $$\mathrm{Con}{\left(X_{\mathrm{ik}}\right)}_{\mathrm{LGC}}=\frac{\lambda_{\mathrm{ik}}^{2}\;\gamma_{i}^{2}\;\mathrm{Var}\left(T\right)+\lambda_{\mathrm{ik}}^{2}\;\sigma_{i}^{2}\;\mathrm{Var}\left(\mathrm{SL}\right)+{2\;\lambda }_{\mathrm{ik}}^{2}\;\gamma_{i}\;\sigma_{i}\;\mathrm{Cov}\left(T,\mathrm{SL}\right)}{\mathrm{Var}\left(X_{\mathrm{ik}}\right)}$$

Occasion specificity is defined as the proportion of variance in manifest variables due to the situation and the person × situation interaction:

(4) $$\mathrm{Spe}\left(X_{\mathrm{ik}}\right)=\frac{\lambda_{\mathrm{ik}}^{2}\;\mathrm{Var}\left({\mathrm{SR}}_{i}\right)}{\mathrm{Var}\left(X_{\mathrm{ik}}\right)}$$

#### 2.6.2 Internal Consistency and Temporal Stability

In addition, we aimed to provide traditionally used measures of reliability in order to compare our results to previous studies (e.g., Bargary et al. 2017; Ettinger et al. 2003; Schröder et al. 2021). Cronbach's alpha (Cronbach 1951) was used as a measure of internal consistency. Calculation was based on aggregated SPEM data per half‐cycle. While an equal number of half‐cycles is required to determine Cronbach's alpha, the number of available half‐cycles was not identical for all participants due to different amount of data loss (e.g., due to longer lasting periods of blinks). Hence, we first excluded participants with less than 30 available half‐cycles, as well as outliers on the lower end regarding the number of available half‐cycles (i.e., < mean‐4*IQR). Then, the number of half‐cycles used for Cronbach's alpha was determined based on the participant with the smallest number of available half‐cycles. For participants with more available half‐cycles, the previously determined number was randomly drawn from their available half‐cycles. For intra‐individual standard deviation of RMSE, we determined split‐half reliability instead of Cronbach's alpha, since this variable could not be calculated per half‐cycle. Split‐half reliability was calculated as Spearman correlation based on odd‐even split of cycles.

Temporal stability was assessed based on aggregated SPEM data for each measurement occasion. We calculated two‐way mixed, single measures intraclass correlation (ICC) coefficients with absolute agreement (Bartko 1966, 1991; Koo and Li 2016; McGraw and Wong 1996). Moreover, one‐way repeated‐measures analyses of variance (ANOVAs) with the within‐subjects factor measurement occasion (T1, T2, T3) were calculated for all SPEM variables, calculating partial eta squared ($\eta_{p}^{2}$) for effect sizes (Cohen 1973). If Mauchly's test indicated violation of the assumption of sphericity, Greenhouse–Geisser corrected *p*‐values were determined. Bonferroni‐corrected post hoc *t*‐tests were applied in case of a significant main effect of occasion. Effect sizes for post hoc *t*‐tests were quantified using Cohen's *d* (Cohen 1988). A significance level of 5% was used for all analyses.

## 3 Results

Analyses were based on a final sample of *N* = 163 participants (81 women, 82 men) with a mean age of 22.56 years (SD = 3.01). All participants were university students, 44 (26.99%) of whom studied psychology. Data on all three measurement occasions were available for 146 (89.57%) participants. Descriptive statistics are presented in Table 2.

**TABLE 2:** Descriptive statistics.

	*N*	T1		T2		T3		ICC (95%‐CI)
		*M* (SD)	*α*	*M* (SD)	*α*	*M* (SD)	*α*	
Sinusoidal SPEM								
Velocity gain	162	89.01 (9.43)	0.98	89.99 (7.84)	0.98	89.76 (7.80)	0.98	0.68 (0.60–0.75)
SD of velocity gain	163	23.71 (4.83)	0.98	23.20 (4.52)	0.97	23.54 (5.31)	0.98	0.53 (0.44–0.62)
RMSE	159	1.51 (0.76)	0.98	1.41 (0.60)	0.97	1.49 (0.77)	0.98	0.60 (0.51–0.68)
SD of RMSE	161	0.81 (0.63)	0.78^a^	0.76 (0.58)	0.66^a^	0.77 (0.60)	0.73^a^	0.55 (0.46–0.64)
Saccade frequency	163	1.09 (0.47)	0.96	1.06 (0.43)	0.95	1.07 (0.47)	0.96	0.79 (0.73–0.83)
Triangular SPEM								
Velocity gain	162	93.77 (6.80)	0.97	94.42 (6.02)	0.98	94.54 (6.14)	0.98	0.69 (0.62–0.76)
SD of velocity gain	163	30.14 (6.72)	0.97	29.73 (6.15)	0.97	30.65 (6.69)	0.97	0.61 (0.52–0.69)
RMSE	161	1.50 (0.64)	0.98	1.53 (0.65)	0.98	1.62 (0.82)	0.98	0.61 (0.52–0.69)
SD of RMSE	160	0.64 (0.41)	0.68^a^	0.61 (0.39)	0.73^a^	0.65 (0.41)	0.70^a^	0.58 (0.49–0.66)
Saccade frequency	163	1.05 (0.39)	0.95	1.00 (0.37)	0.95	1.00 (0.39)	0.95	0.77 (0.71–0.82)

*Note:* Means and standard deviations are shown for each variable per measurement occasion (T1, T2, T3). Mean and intra‐individual standard deviation of velocity gain are given in percent (%), mean and intra‐individual standard deviation of root mean square error (RMSE) are given in degrees of visual angle (°) and saccade frequency refers to the number of saccades per second (N/s). For test–retest reliability, we present intraclass correlation coefficients (ICC). For internal consistency, we present Cronbach's alpha (*α*), except for intra‐individual standard deviation of RMSE, where we present split‐half reliability as Spearman correlation based on odd‐even split of cycles, since this variable could not be calculated per half‐cycle.

Abbreviation: SPEM, smooth pursuit eye movement.

^a^ Numbers represent split‐half reliability (ρ_odd,even_) instead of Cronbach's *α*.

### 3.1 SEM Results

Model parameters are shown in Table 3. All variables could be modeled without requiring indicator‐specific factors. At least strong MI was given for all variables, except for SD of velocity gain, RMSE and SD of RMSE in the triangular SPEM task (Table S1). Thus, models were set up using three instead of two indicators for these variables (for two indicator models see Table S2). While at least strong MI was given for SD of velocity gain and SD of RMSE when using three indicators, this was not the case for RMSE (Table S1). Nevertheless, model fits for RMSE were generally better when using three compared to two indicators and LST/LGC models yielded reasonable model estimates. As using four indicators did not lead to MI either (Table S1) we report results based on models using three indicators. However, results for RMSE in the triangular SPEM task should be interpreted with caution.

**TABLE 3:** Model parameters.

	Model	Restriction	*p*_compare_	*χ* ^2^ (df, *p*)	CFI	RMSEA (95% CI, *p*)	SRMR
Sinusoidal SPEM							
Velocity gain (*N* = 162)	LST^a^			19.08 (12, 0.087)	1.00	0.06 (0.00–0.14, 0.358)	0.02
	**LST**_**T**_	**A**	**0**.**303**	**25.34 (18, 0.116)**	**0**.**99**	**0**.**06 (0.00–0.13, 0.355)**	**0**.**06**
	LGC	C	0.204	28.68 (19, 0.071)	0.99	0.06 (0.00–0.12, 0.383)	0.03
SD of velocity gain (*N* = 163)	LST	C	0.464*	17.28 (21, 0.694)	1.00	0.00 (0.00–0.06, 0.915)	0.07
	**LST**_**T**_	**C**	**0**.**707**	**18.16 (23, 0.749)**	**1.00**	**0**.**00 (0.00–0.05, 0.942)**	**0**.**07**
	LGC	A		12.45 (16, 0.712)	1.00	0.00 (0.00–0.06, 0.915)	0.02
RMSE (*N* = 159)	LST	A		18.85 (16, 0.277)	1.00	0.00 (0.00–0.10, 0.689)	0.03
	LST_T_	A	**0**.**671**	**19.8 (18, 0.344)**	**1.00**	**0**.**00 (0.00–0.09, 0.759)**	**0**.**04**
	LGC	A		18.85 (16, 0.277)	1.00	0.00 (0.00–0.10, 0.689)	0.03
SD of RMSE (*N* = 161)	LST			14.02 (11, 0.232)	1.00	0.00 (0.00–0.11, 0.666)	0.03
	**LST**_**T**_		**0**.**661**	**15 (13, 0.308)**	**1.00**	**0**.**00 (0.00–0.09, 0.742)**	**0**.**04**
	LGC			14.02 (11, 0.232)	1.00	0.00 (0.00–0.11, 0.666)	0.03
Saccade frequency (*N* = 163)	LST	B	0.728*	25.96 (20, 0.167)	0.99	0.05 (0.00–0.09, 0.506)	0.07
	**LST**_**T**_	**B**	**0**.**440**	**27.71 (22, 0.186)**	**1.00**	**0**.**04 (0.00–0.09, 0.552)**	**0**.**07**
	LGC^b^	B		28 (19, 0.083)	0.99	0.06 (0.00–0.10, 0.374)	0.06
Triangular SPEM							
Velocity gain (*N* = 162)	LST	B	0.035^†^	15.08 (20, 0.772)	1.00	0.00 (0.00–0.06, 0.927)	0.06
	**LST**_**T**_	**A**		**9.3 (18, 0.952)**	**1.00**	**0**.**00 (0.00–0.00, 0.988)**	**0**.**03**
	LGC^c^	C		13.79 (20, 0.841)	1.00	0.00 (0.00–0.05, 0.958)	0.02
SD of velocity gain (*N* = 163)	LST^e^	C	0.466*	51.33 (48, 0.345)	1.00	0.02 (0.00–0.07, 0.818)	0.08
	**LST**_**T**_ ^e^	**C**	**0**.**184**	**54.41 (50, 0.310)**	**1.00**	**0**.**02 (0.00–0.07, 0.803)**	**0**.**08**
	LGC^e^	C		50.68 (46, 0.294)	1.00	0.02 (0.00–0.07, 0.822)	0.04
RMSE (*N* = 161)^d^	LST^e^			57.11 (34, 0.008)	0.99	0.07 (0.00–0.12, 0.299)	0.07
	**LST**_**T**_ ^e^		**0**.**693**	**56.34 (36, 0.017)**	**0**.**99**	**0**.**06 (0.00–0.11, 0.368)**	**0**.**07**
	LGC^e^			57.11 (34, 0.008)	0.99	0.07 (0.00–0.12, 0.299)	0.07
SD of RMSE (*N* = 160)	LST^e^	A		56.66 (42, 0.065)	0.97	0.06 (0.00–0.10, 0.343)	0.07
	**LST**_**T**_ ^e^	**A**	**0**.**233**	**59.52 (44, 0.059)**	**0**.**97**	**0**.**06 (0.00–0.10, 0.347)**	**0**.**09**
	LGC^e^	B	0.404	58.74 (44, 0.068)	0.97	0.06 (0.00–0.10, 0.365)	0.08
Saccade frequency (*N* = 163)	LST	C	0.042* 0.049^†^	19.45 (21, 0.556)	1.00	0.00 (0.00–0.07, 0.843)	0.03
	**LST**_**T**_	**A**		**9.93 (18, 0.934)**	**1.00**	**0**.**00 (0.00–0.02, 0.988)**	**0**.**05**
	LGC	C		10.75 (19, 0.932)	1.00	0.00 (0.00–0.02, 0.990)	0.02

*Note:* The respective most restrictive best fitting models are shown for latent state–trait models allowing variations in state intercepts (LST) or not (LST_T_) as well as for latent growth curve (LGC) models, respectively. The most restrictive best fitting model for each variable is highlighted in bold, based on model fit indices as well as model comparisons using *χ* ^2^ difference tests (*p*_compare_; LST_T_ and LGC models compared to LST model). We used robust maximum likelihood estimation (MLR, including robust standard errors and scaled test statistics) for all variables due to violation of multivariate normality. A = equal measurement error variances; B = equal measurement error variances, equal state residuals, trait loadings fixed to 1; C = equal measurement error variances, equal state residuals, trait and state loadings fixed to 1.

Abbreviations: CFI, comparative fit index; RMSE, root mean square error; RMSEA, root mean square error of approximation; SPEM, smooth pursuit eye movement; SRMR, standardized root mean residual.

^a^ Measurement error variance for odd cycles on the second occasion was fixed to 0 since it was negative.

^b^ Slope variance was negative and thus, the slope loading for the third measurement occasion was fixed to the freely estimated loading (*σ*_3_ = 1.36) from a model with equal measurement variances.

^c^ Slope variance was negative and thus, the slope loading for the third measurement occasion was fixed to the freely estimated loading (*σ*_3_ = 0.92) from a model with equal measurement variances.

^d^ At least strong measurement invariance was not given.

^e^ Models were based on three indicators rather than two.

* *p*‐value refers to the comparison between LGC and LST models.

^†^ *p*‐value refers to the comparison between LST_T_ and LST models.

Since LT models generally had a poor fit (Table S3), we did not further consider these models. Model fits were excellent and similar between LST, LST_T_ and LGC models for most variables. However, *χ* ^2^ difference test indicated that the LST model fit significantly worse than the LST_T_ model for velocity gain (*p* = 0.035) and saccade frequency (*p* = 0.049) in the triangular SPEM task. For saccade frequency, the LST model also fit significantly worse than the LGC model (*p* = 0.042). In addition, model fit was not optimal for RMSE in the triangular SPEM task (χ^2^ was significant in all models, *p* < 0.018; RMSEA = 0.07).

Overall, model fit between LST and LST_T_ models was similar, or LST models fit significantly worse. Thus, latent state trait models not allowing for variations in state intercepts (i.e., LST_T_) were selected as most restrictive best fitting latent state trait models (see Table 4 for model estimates).

**TABLE 4:** Model estimates.

	Model	T Mean (SE)	S_1_ Intercept (SE)	S_2_ Intercept (SE)	S_3_ Intercept (SE)	SL Mean (SE)	SL SD (SE)	*r* (T, SL)
			*σ*_1_ Slope loading	*σ*_2_ Slope loading	*σ*_3_ Slope loading			
Sinusoidal SPEM								
Velocity gain	**LST**_**T**_	**89.07** * **(0.73)**						
	LGC	89.02* (0.77)	*0*	*1*	1.09*	0.66 (0.59)	3.33 (3.33)	−0.644
SD of velocity gain	**LST**_**T**_	**23.49** * **(0.33)**						
	LGC	23.59* (0.38)	*0*	*1*	0.45	−0.31 (0.35)	2.35 (4.13)	−0.130
RMSE	**LST**_**T**_	**1.51** * **(0.06)**						
	LGC	1.51* (0.06)	*0*	*1*	0.54	−0.1* (0.05)	0.35 (0.4)	−0.502
SD of RMSE	**LST**_**T**_	**0.77** * **(0.05)**						
	LGC	0.79* (0.05)	*0*	*1*	0.92	−0.06 (0.05)	0.35 (0.92)	−0.573
Saccade frequency	**LST**_**T**_	**1.07** * **(0.03)**						
	LGC	1.08* (0.04)	*0*	*1*	*1.36*	−0.02 (0.02)	0 (0.1)	−0.234
Triangular SPEM								
Velocity gain	**LST**_**T**_	**93.65** * **(0.53)**						
	LGC	93.74* (0.53)	*0*	*1*	*0.92*	0.7 (0.4)	2.92* (1.93)	−0.513*
SD of velocity gain	**LST**_**T**_	**30.11** * **(0.44)**						
	LGC	30.33* (0.5)	*0*	*1*	0.16	−0.58 (0.54)	2.81 (3.5)	−0.488
RMSE^a^	**LST**_**T**_	**1.52** * **(0.06)**						
	LGC	1.53* (0.06)	*0*	*1*	4.48	0.02 (0.05)	0 (0.1)	0.265
SD of RMSE	**LST**_**T**_	**0.61** * **(0.04)**						
	LGC	0.63* (0.04)	*0*	*1*	0.86*	−0.01 (0.03)	0.22 (0.17)	−0.356
Saccade frequency	**LST**_**T**_	**1.04** * **(0.03)**						
	LGC	1.05* (0.03)	*0*	*1*	1*	−0.05* (0.02)	0.17* (0.1)	−0.310

*Note:* Model estimations for all variables are shown based on the respective most restrictive best fitting latent state trait (without varying state intercepts; LST_T_) and latent growth curve (LGC) models. The most restrictive best fitting model for each variable is highlighted in bold. Fixed values are shown in italics.

Abbreviations: *σ*_1‐3_, slope loadings; RMSE, root mean square error; S_1‐3_, latent states; SL, latent slope; SPEM, smooth pursuit eye movement; T, latent trait.

* *p* < 0.05.

^a^ At least strong measurement invariance was not given.

LGC model estimates (Table 4) suggested that there were mostly no significant trait changes or individual differences in trait changes over time (slope mean and standard deviation mostly n.s.). For RMSE in the sinusoidal SPEM task and saccade frequency in the triangular task, slope means were significant but negligible in size, indicating a change between measurement occasions one and two of −0.1 points (RMSE) and −0.05 points (saccade frequency), respectively. Repeated measures ANOVAs revealed a similar pattern, yielding no significant main effect of measurement occasion, except for a significant effect on saccade frequency in the triangular SPEM task. However, post hoc *t*‐tests indicated no significant pairwise differences between measurement occasions after Bonferroni‐correction (see Table 5 and Figures S1 and S2 for ANOVA and *t*‐test results). In addition, LGC model estimates showed significant individual differences in trait changes for velocity gain and saccade frequency in the triangular SPEM task reflected by significant slope standard deviations (velocity gain: 2.92; saccade frequency: 0.17). For velocity gain, there was also a significant negative correlation between trait and slope (*r* = −0.513), indicating that individuals with high trait scores had less improvement on trait level over time (however, slope mean = 0.7 and n.s.).

**TABLE 5:** Repeated measures effects.

	Effect	*N*	df_n_	df_d_	*F*	*p*	$\eta_{p}^{2}$ [95% CI]	𝜖
Sinusoidal SPEM								
Velocity gain	Occasion	143	2	284	1.08	0.339	0.008 [0.000, 0.034]	
SD of velocity gain	Occasion	142	2	282	0.52	0.597	0.004 [0.000, 0.024]	
RMSE	Occasion	141	2	280	2.19	0.114	0.015 [0.000, 0.051]	
SD of RMSE	Occasion	144	2	286	0.75	0.475	0.005 [0.000, 0.028]	
Saccade frequency	Occasion	146	2	290	0.64	0.529	0.004 [0.000, 0.026]	
Triangular SPEM								
Velocity gain	Occasion	144	2	286	1.24	0.290	0.009 [0.000, 0.037]	0.95
SD of velocity gain	Occasion	146	2	290	1.66	0.192	0.011 [0.000, 0.042]	
RMSE	Occasion	139	2	276	1.41	0.245	0.010 [0.000, 0.041]	0.95
SD of RMSE	Occasion	139	2	276	1.53	0.220	0.011 [0.000, 0.042]	0.95
Saccade frequency	Occasion	146	2	290	3.38	**0**.**039** *	0.023 [0.000, 0.063]	0.93

*Note:* Greenhouse–Geisser epsilon (𝜖) and corrected *p*‐values are given for analyses in which sphericity was not given. Significant effects are highlighted in bold.

* While uncorrected post hoc *t*‐tests indicated that saccade frequency was higher at measurement occasion one (T1) compared to occasion three (T3) (*p* = 0.025) and marginally higher at T1 than at occasion two (T2) (*p* = 0.056), none of these effects survived correcting for multiple testing; T1 vs. T2: *t*(145) = 1.92, *p* = 0.169, *d* = 0.159; T1 vs. T3: *t*(145) = 2.27, *p* = 0.074, *d* = 0.188. There was no significant difference in saccade frequency between T2 and T3, *t*(145) = 0.39, *p* = 1.000, *d* = 0.032.

While both LST_T_ and LGC models had excellent fit, LGC model estimates mostly did not indicate meaningful trait changes or individual differences in trait changes. Thus, calculations of model‐based reliability, consistency, and occasion specificity were based on LST_T_ models, also regarding intra‐individual standard deviation of RMSE in triangular SPEM despite SRMR not meeting the threshold of ≤ 0.08 (SRMR = 0.09).

### 3.2 Reliability, Consistency and Occasion Specificity

**FIGURE 2:**
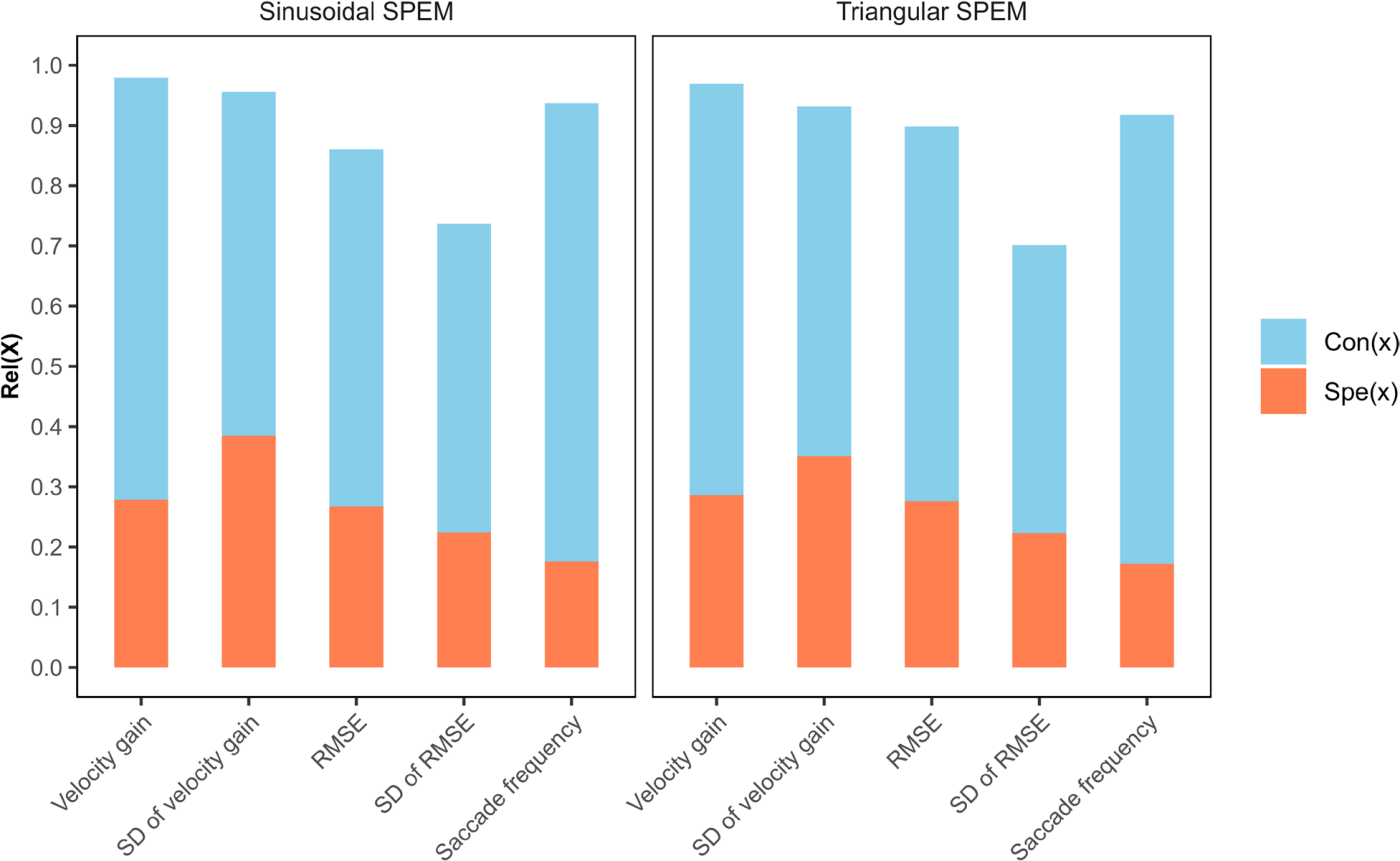
Reliability, Consistency and Occasion Specificity. Reliability [Rel(X)], consistency [Con(X)], and occasion specificity [Spe(X)] of each variable are illustrated, referring to average values across measurement occasions and test sets. At least strong measurement invariance was not given for RMSE (root mean square error) in the triangular SPEM (smooth pursuit eye movement) task.

Model‐based reliability (Rel), consistency (Con) and occasion specificity (Spe) are presented in Table 6 (separately per measurement occasion and test set, as well as averaged values) and in Figure 2 (averaged values). Internal consistency and temporal stability are given in Table 2.

**TABLE 6:** Reliability, consistency and occasion specificity.

		M	T1	T2	T3
Sinusoidal SPEM					
Velocity gain (*N* = 162)	Rel(x)	0.98	0.98/0.98	0.98/0.98	0.98/0.98
	Con(x)	0.70	0.61/0.61	0.79/0.79	0.71/0.71
	Spe(x)	0.28	0.38/0.38	0.19/0.19	0.27/0.27
SD of velocity gain (*N* = 163)	Rel(x)	0.96	0.96/0.96	0.96/0.96	0.96/0.96
	Con(x)	0.57	0.57/0.57	0.57/0.57	0.57/0.57
	Spe(x)	0.38	0.38/0.38	0.38/0.38	0.38/0.38
RMSE (*N* = 159)	Rel(x)	0.86	0.88/0.88	0.83/0.83	0.87/0.87
	Con(x)	0.59	0.56/0.56	0.67/0.67	0.55/0.55
	Spe(x)	0.27	0.32/0.32	0.16/0.16	0.32/0.32
SD of RMSE (*N* = 161)	Rel(x)	0.74	0.81/0.87	0.75/0.65	0.53/0.81
	Con(x)	0.51	0.48/0.51	0.54/0.47	0.43/0.65
	Spe(x)	0.22	0.34/0.36	0.21/0.18	0.10/0.16
Saccade frequency (*N* = 163)	Rel(x)	0.94	0.94/0.94	0.94/0.94	0.94/0.94
	Con(x)	0.76	0.76/0.76	0.76/0.76	0.76/0.76
	Spe(x)	0.18	0.18/0.18	0.18/0.18	0.18/0.18
Triangular SPEM					
Velocity gain (*N* = 162)	Rel(x)	0.97	0.97/0.97	0.97/0.97	0.97/0.97
	Con(x)	0.68	0.59/0.59	0.72/0.72	0.74/0.74
	Spe(x)	0.29	0.38/0.38	0.25/0.25	0.23/0.23
SD of velocity gain (*N* = 163)	Rel(x)	0.93	0.93/0.93/0.93	0.93/0.93/0.93	0.93/0.93/0.93
	Con(x)	0.58	0.58/0.58/0.58	0.58/0.58/0.58	0.58/0.58/0.58
	Spe(x)	0.35	0.35/0.35/0.35	0.35/0.35/0.35	0.35/0.35/0.35
RMSE (*N* = 161)^a^	Rel(x)	0.90	0.92/0.89/0.91	0.90/0.89/0.93	0.82/0.96/0.85
	Con(x)	0.62	0.63/0.61/0.63	0.71/0.70/0.73	0.50/0.59/0.52
	Spe(x)	0.28	0.29/0.28/0.29	0.20/0.20/0.21	0.32/0.37/0.33
SD of RMSE (*N* = 160)	Rel(x)	0.70	0.74/0.69/0.75	0.70/0.65/0.71	0.71/0.66/0.72
	Con(x)	0.48	0.43/0.41/0.44	0.49/0.46/0.50	0.53/0.50/0.54
	Spe(x)	0.22	0.30/0.29/0.31	0.21/0.19/0.21	0.17/0.16/0.17
Saccade frequency (*N* = 163)	Rel(x)	0.92	0.93/0.93	0.91/0.91	0.91/0.91
	Con(x)	0.75	0.68/0.68	0.78/0.78	0.77/0.77
	Spe(x)	0.17	0.24/0.24	0.13/0.13	0.14/0.14

*Note:* Reliability [Rel(X)], consistency [Con(X)], and occasion specificity [Spe(X)] of each variable are shown separately per test set (odd/even or set1/set2/set3) and measurement occasion (T1, T2, T3), as well as average values (*M*).

Abbreviations: RMSE, root mean square error; SPEM, smooth pursuit eye movement.

^a^ At least strong measurement invariance was not given.

Model‐based reliability and internal consistency were mostly excellent (Rel = 0.86–0.98, Cronbach's *α* = 0.95–0.98), except for intra‐individual standard deviation of RMSE in both SPEM tasks where values were good (Rel = 0.70–0.74, *ρ*_odd,even_ = 0.66–0.78). ICC values ranged from moderate to good (ICC = 0.53–0.79) and tended to be smaller for variables depicting intra‐individual variability compared to other variables.

Consistency (Con = 0.48–0.76) was generally higher than occasion specificity (Spe = 0.17–0.38), indicating that a higher proportion of variance was due to trait influences (on average 62%) than due to influences of the situation and person × situation (on average 26%). To further examine this pattern, we quantified the amount of explained variance that can be attributed to trait influences (Con/Rel × 100). On average, about 70% of explained variance was due to trait influences, with highest trait influences observed for saccade frequency (sinusoidal SPEM: 81%, triangular SPEM: 82%) and velocity gain (sinusoidal SPEM: 71%, triangular SPEM: 70%).

## 4 Discussion

While previous studies have shown mostly good reliability of SPEM performance (Table 1), the relative amounts of trait and state influences have not yet been dismantled. However, knowledge about relative amounts of variance from both sources is crucial to different lines of SPEM research, from experimental to clinical and individual differences studies. The present study aimed to address this research gap by applying LST and LGC modeling to SPEM for the first time. To do so, SPEM tasks with sinusoidal and triangular movement patterns were presented at three measurement occasions, using a sample of *N* = 163 healthy university students. We expected at least good reliability and larger trait compared to state influences on SPEM performance.

Our main findings were as follows. First, we found mostly excellent model‐based reliability of SPEM performance. Model‐based reliability of the intra‐individual standard deviation of RMSE in both tasks was lower, but good. *Second*, while trait influences were generally higher than state influences, state influences were also substantial. Third, mostly no or only negligible trait changes could be observed in LGC models, and for all variables, LST_T_ models were selected as the basis to calculate model‐based reliability, consistency and occasion specificity. Fourth, LST, LST_T_ and LGC models had good fits while LT models generally did not fit, showing that SPEM performance could successfully be modeled based on assumptions from LST theory. Model‐based results for RMSE in the triangular SPEM task should be interpreted with caution due to lack of at least strong MI.

### 4.1 Reliability

As expected, SPEM performance was highly reliable (Rel = 0.70–0.98), suggesting that measurement error was small. Concerning traditional CTT‐based reliability estimations, temporal stability (ICC = 0.53–0.79) was mostly comparable but tended to be on the upper end of what has been reported previously, while internal consistency (Cronbach's *α* = 0.95–0.98, *ρ*_odd,even_ = 0.66–0.78) was mostly larger than in previous reports (Table 1). Crucially, LST model‐based reliability coefficients were larger compared to temporal stability reported here, as well as previous reports based on CTT.

While traditional reliability measures do not explicitly differentiate between trait and situational influences, these two sources of variance can be decomposed and quantified using measures based on LST theory (Geiser et al. 2015; Steyer et al. 1999, 2015). Given substantial situational influences on SPEM performance in our study, and in line with what has been pointed out previously (Steyer et al. 2015), our findings suggest that a substantial part of what is attributed to measurement error in longitudinal designs using CTT might be considered as situational fluctuations in reliability estimations based on LST theory. Thus, the underlying test theory might have an impact on which variance components are attributed to measurement error, thereby influencing reliability estimations.

### 4.2 Trait and State Influences

In line with our hypothesis, we found higher consistency (Con = 0.48–0.76) than occasion specificity (Spe = 0.17–0.38), reflecting larger trait influences than influences of the situation and the person × situation interaction on SPEM performance. The pattern of higher trait than state influences is consistent with previous LST studies on prosaccades, antisaccades and memory‐guided saccades (Faßbender et al. 2023; Con = 0.77–0.82, Spe = 0.07–0.12; Meyhöfer et al. 2016; Con = 0.22–0.98, Spe = −0.08–0.24). However, this pattern was less pronounced in our study since we also observed substantial state influences. Crucially, Faßbender et al. (2023) used the same sample as in our study and found much smaller situational influences for prosaccades and antisaccades compared to our findings for SPEM. This suggests substantial situational influences particularly for SPEM, and less so for saccadic eye movements. Moreover, we found that LT models could not adequately model SPEM performance, further stressing the relevance to additionally include latent states and state residuals to be able to model situational fluctuations.

Based on our findings, the question regarding potential sources of situational variance on SPEM performance arises, particularly in contrast to saccadic eye movements. However, these are difficult to determine since we already controlled for several factors. First, we defined the presence of any current or past psychiatric condition as well as current medication intake as exclusion criteria since these factors have been shown to be associated with SPEM performance (Roy‐Byrne et al. 1993; Schmechtig et al. 2013; Smyrnis et al. 2019). Moreover, measurement occasions were arranged to be exactly 1 week apart at the same time of day to prevent time of day effects (Roy‐Byrne et al. 1995; Schalén et al. 1983). Potential time‐on‐task effects on SPEM (Březinová and Kendell 1977) were also controlled for since tasks were always presented at the beginning of a session and only took about 3 min.

One potential source of situational fluctuations we did not control for is sleepiness, and in its extreme form sleep deprivation, which however affects both SPEM and saccadic eye movements (De Gennaro et al. 2001; Meyhöfer et al. 2017). Additionally, there is evidence of deteriorated SPEM and saccadic performance under cognitive distraction (i.e., secondary tasks; Korda et al. 2023; Meyer et al. 2007), which might have played a role in our study in the form of other internally directed cognitive aspects such as distracting thoughts. Overall, due to similar effects of these situational factors on both SPEM and saccades, it is unclear why situational fluctuations for SPEM were larger compared to saccades.

While situational variability reflects short‐term, situation‐driven fluctuations, trait changes refer to longer‐lasting modifications on trait level (e.g., learning processes; Steyer et al. 2015). In contrast, trait and situational variance cannot be decomposed in traditional repeated measures analyses. While there were substantial situational fluctuations of SPEM, performance was relatively stable over time as indicated by mostly no or only negligible trait changes. In addition, we did not find evidence for substantial changes in performance using traditional repeated measures analyses, which is in line with previous research (Ettinger et al. 2003; Flechtner et al. 2002; Gooding et al. 1994; Schröder et al. 2021). This is in contrast to findings by Faßbender et al. (2023), where LGC models indicated significant trait changes over time in antisaccade and prosaccade performance. This suggests that while situational influences play a larger role in SPEM compared to saccadic eye movements, trait components of SPEM performance are more stable.

### 4.3 Implications

By applying LST theory, we extend existing literature on the reliability of SPEM by explicitly decomposing proportions of trait and state variance. Our findings of generally high reliability, larger trait but also substantial state influences, and mostly no trait changes indicate that SPEM performance is mainly influenced by stable trait components, but is also influenced by situational fluctuations. Overall, this supports the suitability of SPEM for use in individual differences research. Moreover, our results are in line with the endophenotype status of SPEM by providing evidence on the fulfillment of criteria regarding reliable measurement (Liu and Gershon 2024) and trait‐like nature (Glahn et al. 2014; Gottesman and Gould 2003) in a healthy sample.

The relevance of situational fluctuations in SPEM performance suggests that future studies should more explicitly consider such state influences. Consistent with assumptions from LST theory (Steyer et al. 2015), we showed that the underlying test theory might have an impact on the interpretation of situational variance. For research particularly interested in trait components of SPEM performance, it would thus be useful to explicitly decompose trait and state variance by applying LST theory. Crucially, this would provide the opportunity to directly assess correlations of trait components between SPEM and other constructs, such as clinically relevant personality correlates. In addition, potential sources of situational fluctuations in SPEM performance should be studied more systematically, especially in direct comparison to saccadic eye movements. Particularly in experimental research, systematic identification of situational factors could lead to better control of these potentially confounding factors.

### 4.4 Limitations

There were some limitations to the present study.

First, for RMSE in the triangular SPEM task, MI was not given and model fits were not optimal. However, at least strong MI is required for proper LST and LGC modeling (Geiser et al. 2015). While model‐based results for that variable should be interpreted with caution, they may still provide valuable information.

In addition, models were based on three measurement occasions with two indicators each. However, it has been recommended to use four or more measurement occasions with at least three indicators each (Geiser et al. 2015). While model‐based reliability, consistency, and occasion specificity may still be estimated sufficiently based on our design (Geiser et al. 2015), overall using more indicators would have been ideal given our sample size (Geiser et al. 2015; Schermelleh‐Engel et al. 2003). This fits the observation that some of the SPEM parameters could be successfully modeled only when using three instead of two indicators per measurement occasion.

Moreover, reliability cannot be regarded as an inherent task characteristic, but always refers to a particular measurement (Parsons et al. 2019). Thus, our results regarding reliability as well as trait and state influences are not generalizable to SPEM tasks in general. For example, it has been shown that the reliability of SPEM performance parameters might depend on task difficulty (Ettinger et al. 2003; Schröder et al. 2021). In addition, other aspects of task design, eye‐tracking methodology, and sample characteristics may play a role. Regarding the latter, our sample of young and healthy university students may be considered relatively homogenous, potentially coinciding with relatively low inter‐individual variability (see Faßbender et al. 2023 who used the same sample).

## 5 Conclusions

Overall, we found that SPEM performance is highly reliable and mainly influenced by stable traits, but also by substantial situational fluctuations. This implicates that there is relatively stable inter‐individual variability in SPEM performance, supporting the use of SPEM in individual differences studies. However, situational fluctuations relating to SPEM are also relevant, even while controlling for aspects such as medication intake or time of day. Hence, future studies examining SPEM should consider to explicitly account for situational fluctuations, for example by applying LST theory. Moreover, potential sources of situational variance in SPEM should be identified systematically.

## Author Contributions

Celina Kullmann: data curation, formal analysis, validation, visualization, writing – original draft, writing – review and editing. Ulrich Ettinger: conceptualization, project administration, resources, supervision, validation, writing – review and editing. Kaja Faßbender: conceptualization, formal analysis, methodology, project administration, supervision, validation, visualization, writing – review and editing.

## Ethics Statement

Ethical approval was obtained by the ethics committee of the Department of Psychology at the University of Bonn.

## Conflicts of Interest

The authors declare no conflicts of interest.

## Supporting information


**Table S1:** Model parameters for latent state models without at least strong measurement invariance.
**Table S2:** Model parameters for two‐indicator models without at least strong measurement invariance.
**Table S3:** Model parameters of latent trait models.
**Figure S1:** Repeated measures effects in the sinusoidal SPEM task.
**Figure S2:** Repeated measures effects in the triangular SPEM task.

## Data Availability

Study aims and procedures were preregistered at https://aspredicted.org/4j6x‐vr3m.pdf. Materials, pre‐processed data, and analysis scripts are available at https://osf.io/fcmyg/overview?view_only=4d42c9ce61514669a278e7336f01009a.

## Endnotes

^1^
Since at least strong MI was not given for one variable when using three indicators, we decided post hoc to also attempt setting up models with four indicators (see Section 2 for details).

^2^
LT models were not statistically compared with LST, LST_T_, and LGC since model fit indices for LT models generally indicated poor fit.
