# Supplementary material for: Latent State–Trait and Latent Growth Curve Modeling of Smooth Pursuit Eye Movements
Source: Psychophysiology. 2026 Apr 24;63(5):e70299. doi: 10.1111/psyp.70299 (PMC13109632; doi:10.1111/psyp.70299)
Supplement: Supplementary file 1 — Table S1: Model parameters for latent state models without at least strong measurement invariance. Table S2: Model parameters for two‐indicator models without at least strong measurement invariance. Table S3: Model parameters of latent trait models. Figure S1: Repeated measures effects in the sinusoidal SPEM task. Figure S2: Repeated measures effects in the triangular SPEM task. [file PSYP-63-e70299-s001.pdf]

Latent State-Trait and Latent Growth Curve Modelling of Smooth Pursuit Eye Movements

Celina Kullmann, Ulrich Ettinger, Kaja Faßbender

Department of Psychology, University of Bonn, Bonn, Germany

Author Note

We have no known conflict of interest to disclose.

Data and analysis scripts can be found online in R Markdown format (https://osf.io/fcmyg/overview?view_only=4d42c9ce61514669a278e7336f01009a).

Correspondence concerning this article should be addressed to Kaja Faßbender, Department of Psychology, University of Bonn, Kaiser-Karl-Ring 9, 53111 Bonn, Germany. Email: kaja.fassbender@uni-bonn.de

Supplementary Material 1: Calibration / Validation Details

A horizontal-vertical 5-point calibration procedure (EyeLink 1000 built-in sequence) with randomized presentation sequence of fixation points and repetition of the first fixation point was used. Calibration fixation points were white (RGB 255, 255, 255) disks (diameter outer target = 0.36°; diameter inner target = 0.09°) shown on black (0, 0, 0) background. Successful calibration was followed by a validation procedure with identical stimulus characteristics. During both calibration and validation procedures, experimenters performed manual acceptance of each fixation point. Validation was considered successful if it was classified as “GOOD” (worst point error <1.5°, average error < 1.0°) by the SR Research algorithm. Validation classified as “FAIR” (range of worst point error: 1.5°–2.0°, average error range: 1.0°–1.5°) was accepted if calibration/validation could not be improved after several reattempts. For drift correction, a light grey (192, 192, 192) disk (diameter = 0.34°; stroke width = 0.11°) was shown on black background (0, 0, 0) at central position (0°, 0°).

Supplementary Table 1

Model Parameters for Latent State Models Without at Least Strong Measurement Invariance.

	Model	Restriction	$p_{compare}$	χ^2 (*df*, *p*)	CFI	RMSEA (95% CI, *p*)	SRMR
SD of velocity gain (*N* = 163)	LS	Weak MI		19.28 (8, .013)	.99	.10 (.03-.16, .091)	.02
	LS	Strong MI	.517	21.73 (11, .027)	.99	.08 (.02-.14, .147)	.02
RMSE (*N* = 161)	LS	Weak MI		52.27 (8, <.001)	.96	.20 (.12-.28, .001)	.04
	LS	Strong MI	.584	53.32 (11, <.001)	.96	.17 (.10-.23, .002)	.04
	LS [3]	Weak MI		48.93 (28, .008)	.99	.07 (.00-.12, .301)	.07
	LS [3]	Strong MI	.231	57.11 (34, .008)	.99	.07 (.00-.12, .299)	.07
	LS [4]	Weak MI		131.95 (57, <.001)	.97	.09 (.05-.13, .048)	.07
	LS [4]	Strong MI	.741	140.49 (66, <.001)	.97	.09 (.05-.12, .065)	.07
SD of RMSE (*N* = 160)	LS	Weak MI		36.02 (8, <.001)	.94	.16 (.10-.24, .005)	.08
	LS	Strong MI	.175	42.6 (11, <.001)	.94	.14 (.09-.20, .004)	.08

Note. Model parameters for latent state (LS) models applying restrictions of weak and strong measurement invariance (MI) are shown for variables where model fit indices and model comparisons using χ^2 difference tests ($p_{compare}$) indicated that the assumption of at least strong MI was not given. This was the case only for variables from the triangular smooth pursuit eye movement (SPEM) task. We used robust maximum likelihood estimation (MLR, including robust standard errors and scaled test statistics) for all variables due to violation of multivariate normality. While χ^2 difference tests were not significant, all LS models depicted in this table had significant χ^2 statistics and RMSEA values were not optimal. Thus, while LS models implementing the requirement of strong MI did not fit significantly worse than LS models implementing weak MI, model fits of depicted LS models were generally not optimal, indicating that the requirement of at least strong MI was not given. CFI = comparative fit index; RMSE = root mean square error; RMSEA = root mean square error of approximation; SRMR = standardised root mean residual.

Supplementary Table 2

Model Parameters for Two-Indicator Models Without at Least Strong Measurement Invariance.

	Model	Restriction	χ^2 (*df*, *p*)	CFI	RMSEA (95% CI, *p*)	SRMR
SD of velocity gain (*N* = 163)	LST	C	31.69 (21, .063)	.99	.06 (.00-.11, .285)	.08
	LST_T	A	30.58 (18, .032)	.99	.07 (.01-.12, .196)	.07
	LGC	A	26.2 (16, .051)	.99	.07 (.00-.12, .230)	.02
	LT 2	A	940.06 (25, <.001)	.09	.55 (.51-.58, <.001)	19.01
RMSE (*N* = 161)	LST	A	45.06 (16, <.001)	.96	.13 (.07-.19, .015)	.04
	LST_T	A	43.72 (18, .001)	.96	.13 (.07-.18, .019)	.06
	LGC	A	45.06 (16, <.001)	.96	.13 (.07-.19, .015)	.04
	LT 2	A	474.18 (25, <.001)	.32	.45 (.41-.49, <.001)	4.42
SD of RMSE (*N* = 160)	LST	B	34.1 (20, .025)	.95	.09 (.04-.15, .092)	.08
	LST_T	B	36.07 (22, .030)	.95	.09 (.03-.14, .110)	.08
	LGC	B	37.84 (18, .004)	.95	.10 (.05-.15, .050)	.08
	LT 2	A	237.89 (25, <.001)	.35	.31 (.27-.35, <.001)	2.08

Note. Model parameters for the respective most restrictive best fitting two-indicator model without measurement invariance (MI; only variables from the triangular task were affected) are presented with their respective model indices for latent state-trait models allowing variations in state intercepts (LST) or not (LST-T) as well as for latent growth curve (LGC) models. In addition, latent trait (LT) models with intercepts of manifest variables set to 0 are shown. We used robust maximum likelihood estimation (MLR, including robust standard errors and scaled test statistics) for all variables due to violation of multivariate normality. A = equal measurement error variances; B = equal measurement error variances, equal state residuals, trait loadings fixed to 1; C = equal measurement error variances, equal state residuals, trait and state loadings fixed to 1; CFI = comparative fit index; RMSE = root mean square error; RMSEA = root mean square error of approximation; SRMR = standardised root mean residual.

Supplementary Table 3

Model Parameters of Latent Trait Models.

	Model	Restriction	χ^2 (df, p)	CFI	RMSEA (95% CI, p)	SRMR
Sinusoidal SPEM						
Velocity gain (*N* = 162)	LT	A	1144.09 (20, <.001)	.10	.71 (.67-.75, <.001)	101.95
SD of velocity gain (*N* = 163)	LT	A	846.17 (20, <.001)	.07	.62 (.59-.66, <.001)	20.60
RMSE (*N* = 159)	LT	B	318.69 (25, <.001)	.34	.37 (.34-.41, <.001)	3.78
SD of RMSE (*N* = 161)	LT	B	177.41 (25, <.001)	.44	.28 (.24-.32, <.001)	1.47
Saccade frequency (*N* = 163)	LT	B	643.68 (25, <.001)	.51	.41 (.38-.44, <.001)	4.88
Triangular SPEM						
Velocity gain (*N* = 162)	LT	A	1046.03 (20, <.001)	.00	.71 (.67-.75, <.001)	196.97
SD of velocity gain (*N* = 163)	LT [3]	B	1330.7 (52, <.001)	.26	.45 (.43-.47, <.001)	18.98
RMSE (*N* = 161) [a]	LT [3]	A	682.29 (44, <.001)	.50	.38 (.35-.41, <.001)	4.10
SD of RMSE (*N* = 160)	LT [3]	A	324.64 (44, <.001)	.51	.25 (.23-.28, <.001)	1.91
Saccade frequency (*N* = 163)	LT	A	617.27 (20, <.001)	.49	.44 (.41-.47, <.001)	6.17

Note. The respective most restrictive best fitting latent trait (LT) models are shown. All LT models had bad model fit. We used robust maximum likelihood estimation (MLR, including robust standard errors and scaled test statistics) for all variables due to violation of multivariate normality. A = intercepts of manifest variables set to 0; B = intercepts of manifest variables set to 0 and equal measurement error variances; CFI = comparative fit index; RMSE = root mean square error; RMSEA = root mean square error of approximation; SPEM = smooth pursuit eye movement; SRMR = standardised root mean residual.

[3] Models were based on three indicators rather than two.

[a] At least strong measurement invariance was not given.

Supplementary Figure 1

Repeated Measures Effects in the Sinusoidal SPEM Task.

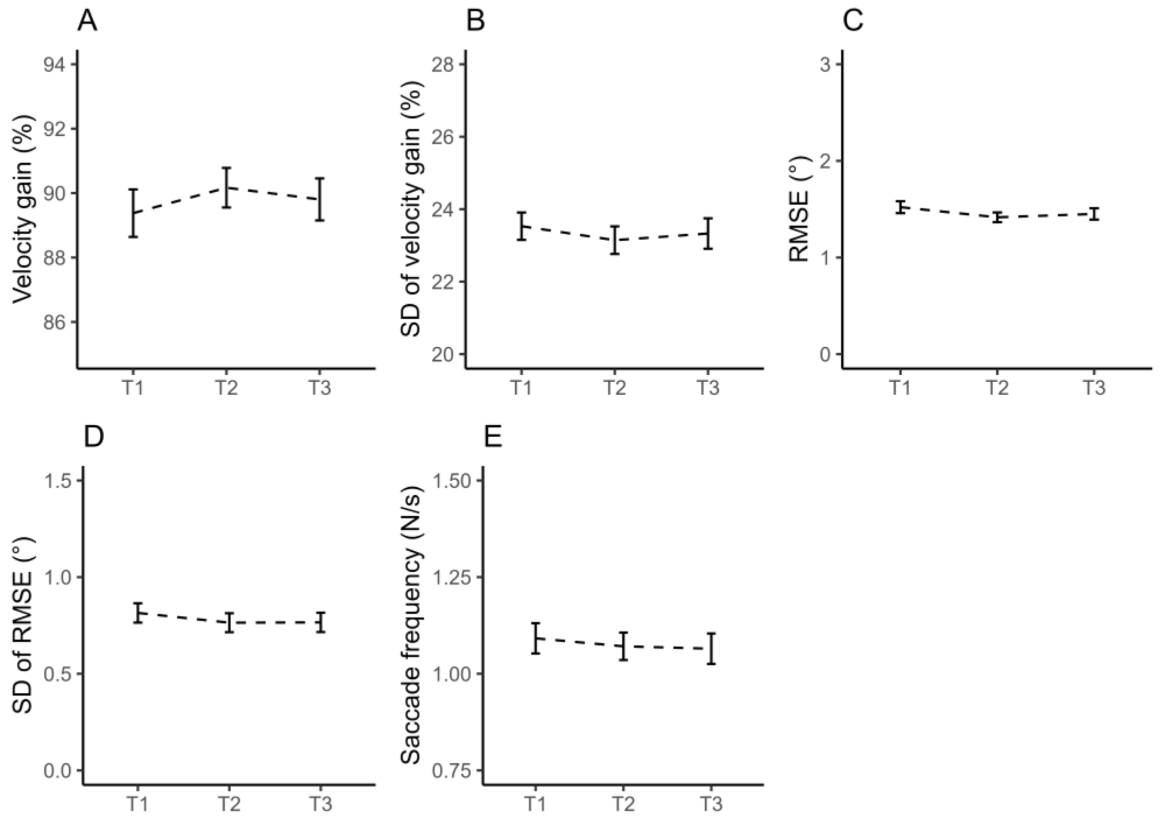

Note. Repeated measures effects are shown graphically for all variables in the sinusoidal SPEM (smooth pursuit eye movement) task. Error bars reflect standard errors. There was no significant effect of measurement occasion. RMSE = root mean square error.

Supplementary Figure 2

Repeated Measures Effects in the Triangular SPEM Task.

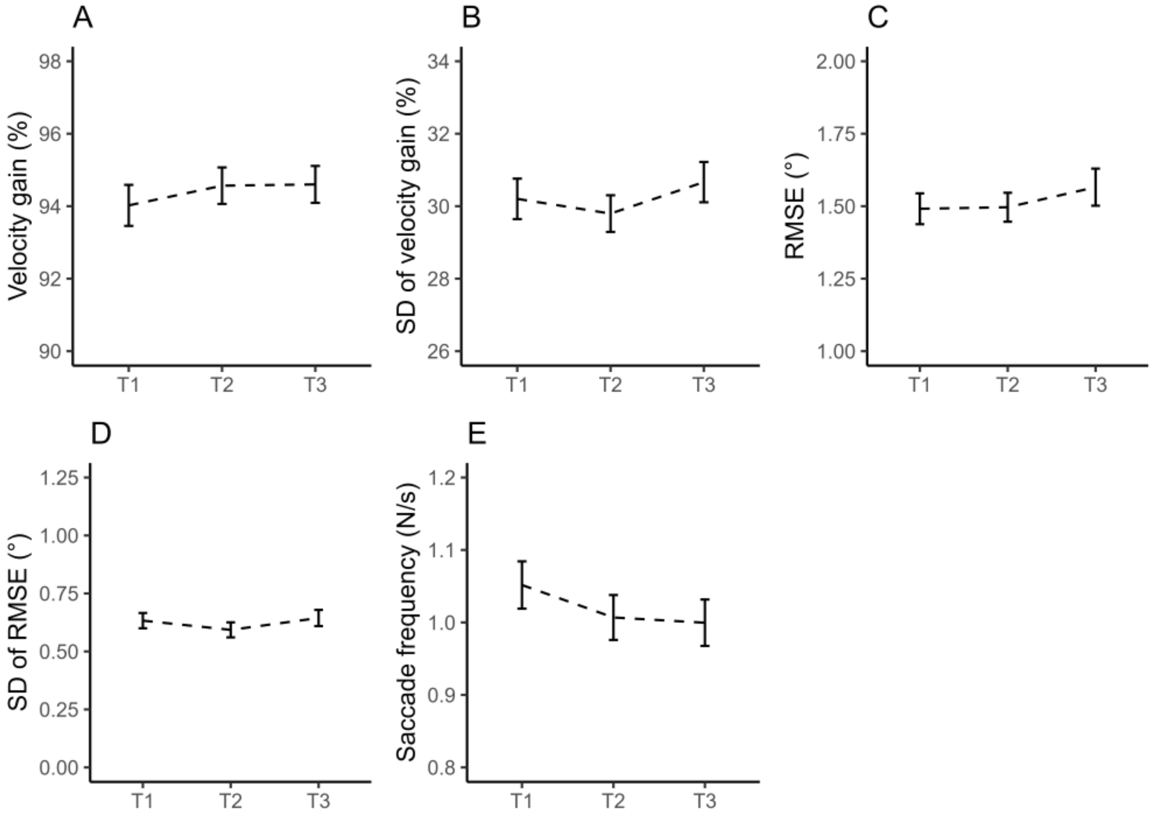

Note. Repeated measures effects are shown graphically for all variables in the triangular SPEM (smooth pursuit eye movement) task. Error bars reflect standard errors. There was a significant effect of measurement occasion only for saccade frequency, but Bonferroni-corrected post-hoc *t*-tests were not significant. RMSE = root mean square error.
